# Survival, Function, and Cognition After Hospitalization in Long-Term Acute Care Hospitals

**DOI:** 10.1001/jamanetworkopen.2024.13309

**Published:** 2024-05-28

**Authors:** Snigdha Jain, Siqi Gan, Oanh K. Nguyen, Rebecca L. Sudore, Michael A. Steinman, Kenneth Covinsky, Anil N. Makam

**Affiliations:** 1 Section of Pulmonary, Critical Care, and Sleep Medicine, Yale School of Medicine, New Haven, Connecticut; 2 Northern California Institute for Research and Education, San Francisco; 3 Division of Geriatrics, Department of Medicine, University of California, San Francisco; 4 Division of Hospital Medicine, San Francisco General Hospital, University of California, San Francisco

## Abstract

**Importance:**

More than 70 000 Medicare beneficiaries receive care in long-term acute care hospitals (LTCHs) annually for prolonged acute illness. However, little is known about long-term functional and cognitive outcomes of middle-aged and older adults after hospitalization in an LTCH.

**Objective:**

To describe survival, functional, and cognitive status after LTCH hospitalization and to identify factors associated with an adverse outcome.

**Design, Setting, and Participants:**

This retrospective cohort study included middle-aged and older adults enrolled in the Health and Retirement Study (HRS) with linked fee-for-service Medicare claims. Included participants were aged 50 years or older with an LTCH admission between January 1, 2003, and December 31, 2016, with HRS interviews available before admission. Data were analyzed between November 1, 2021, and June 30, 2023.

**Main Outcomes and Measures:**

Function and cognition were ascertained from HRS interviews conducted every 2 years. The primary outcome was death or severe impairment in the 2.5 years after LTCH hospitalization, defined as dependencies in 2 or more activities of daily living (ADLs) or dementia. Multivariable logistic regression was performed to evaluate associations with a priori selected risk factors including pre-LTCH survival prognosis (Lee index score), pre-LTCH impairment status, and illness severity characterized by receipt of mechanical ventilation and prolonged intensive care unit stay of 3 days or longer.

**Results:**

This study included 396 participants, with a median age of 75 (IQR, 68-82) years. Of the participants, 201 (51%) were women, 125 (28%) had severe impairment, and 318 (80%) died or survived with severe impairment (functional, cognitive, or both) within 2.5 years of LTCH hospitalization. After accounting for acute illness characteristics, prehospitalization survival prognosis as determined by the Lee index score and severe baseline impairment (functional, cognitive, or both) were associated with an increased likelihood of death or severe impairment in the 2.5 years after LTCH hospitalization (adjusted odds ratio [AOR], 3.2 [95% CI, 1.7 to 6.0] for a 5-point increase in Lee index score; and AOR, 4.5 [95% CI, 1.3 to 15.4] for severe vs no impairment).

**Conclusions and Relevance:**

In this cohort study, 4 of 5 middle-aged and older adults died or survived with severe impairment within 2.5 years of LTCH hospitalization. Better preadmission survival prognosis and functional and cognitive status were associated with lower risk of an adverse outcome, and these findings should inform decision-making for older adults with prolonged acute illness.

## Key Points

**Question:**

What are the longer-term outcomes of middle-aged and older adults after hospitalization in long-term acute care hospitals (LTCHs)?

**Findings:**

In this cohort study with 396 participants, 4 of 5 middle-aged and older adults hospitalized in an LTCH either died or survived with severe impairment characterized by dependencies in 2 or more activities of daily living or dementia within 2.5 years of hospitalization. Better survival prognosis and functional and cognitive status before hospitalization were associated with more favorable outcomes.

**Meaning:**

These findings suggest that most middle-aged and older adults either die or survive with severe impairment (functional, cognitive, or both) within 2.5 years of hospitalization in an LTCH; prehospitalization health could guide patients, families, and clinicians in decisions about prolonged acute care.

## Teaser

This cohort study uses Medicare claims data to assess survival, function, and cognition among middle-aged and older adults after hospitalization in long-term acute care hospitals.

## Introduction

Long-term acute care hospitals (LTCHs) provide multidisciplinary care to medically complex patients who need extended inpatient care after acute care hospitalization.^1,2,3^ Despite recent changes in payment with an overall decline in the number of LTCHs in the US,^4^ more than 70 000 fee-for-service (FFS) Medicare beneficiaries were hospitalized in an LTCH at a cost of $3.4 billion to Medicare.^1^ Mortality rates are high in this medically complex population, with fewer than half of older adults surviving the year after LTCH hospitalization.^5,6^ However, little is known about functional and cognitive outcomes among survivors. These outcomes are important because patients with serious illnesses have goals besides living as long as possible, including maintaining independence.^7^

Understanding of functional and cognitive outcomes in addition to survival is needed to guide goals of care discussions and inform shared decision-making between clinicians and older adults and their families when faced with prolonged acute illness. The few existing studies evaluating long-term functional and cognitive impairments in the LTCH setting are limited in informing outcomes for older adults because of generalizability concerns due to recruiting participants from a small number of LTCHs, recall bias of premorbid assessments of function and cognition, and selection bias from enrollment of patients with less impairment into prospective studies combined with excluding patients with severe debility, which may yield better outcomes.^8,9,10,11,12^ More accurate estimates of long-term functional and cognitive outcomes are necessary because families of patients cared for in an LTCH have overly optimistic expectations of recovery^9,13^ and an unmet need for prognostic information to guide decision-making.^14,15^

In this study, we used data from a longitudinal, nationally representative survey of middle-aged and older adults, the Health and Retirement Study (HRS),^16^ with biennial assessments of function and cognition and linkage to Medicare claims allowing identification of hospitalization in an LTCH. We aimed to describe survival, functional, and cognitive status after LTCH hospitalization and to identify factors associated with an adverse outcome.

## Methods

### Data Source and Study Population

We conducted a retrospective analysis of the HRS, a longitudinal cohort study of US adults aged 50 years or older.^16^ Core interviews were conducted biennially via telephone until death. We used data from the 2002 to 2020 HRS core interviews linked with FFS Medicare claims to identify participants with hospitalization in an LTCH between January 1, 2003, and December 31, 2016. The HRS was approved by the University of Michigan Institutional Review Board. All HRS participants provided informed written consent. The current study was approved by the University of California, San Francisco Institutional Review Board. This report adhered to the Strengthening the Reporting of Observational Studies in Epidemiology (STROBE) reporting guideline.

### Cohort Assembly

**Figure 1.**
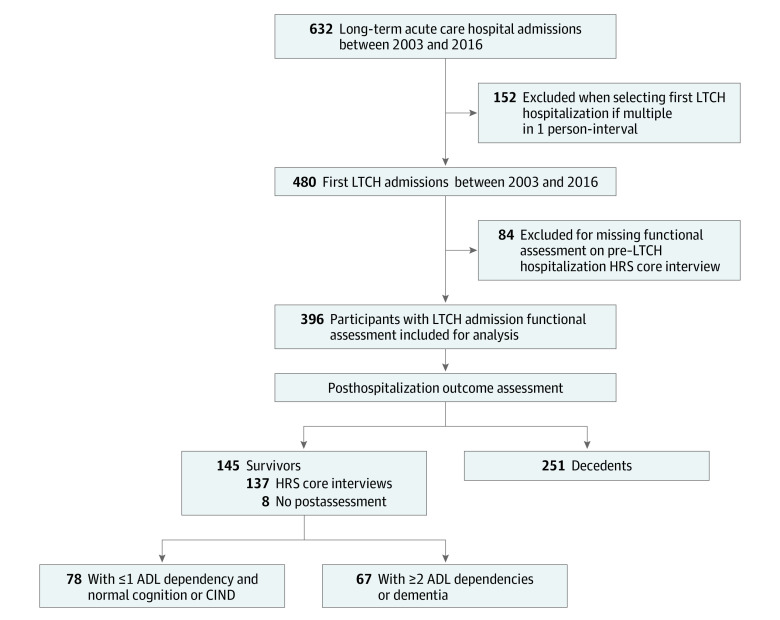
Study Flow Diagram ADL indicates activity of daily living; CIND, cognitive impairment nondementia; HRS, Health and Retirement Study; LTCH, long-term acute care hospital.

We identified 632 LTCH hospitalizations among 480 participants (Figure 1). For participants with multiple stays during the study period, we selected the first hospitalization in the interval between 2 HRS interviews to avoid immortal time bias because survival was an outcome of interest. We excluded participants missing functional or cognitive assessment in the pre-LTCH (baseline) HRS interview, which occurred up to 2.5 years before the LTCH admission, resulting in a sample of 396 participants with an LTCH hospitalization.

### Baseline Characteristics

We ascertained baseline sociodemographic and health characteristics from the HRS core interview immediately preceding the index LTCH hospitalization (hereafter, pre–LTCH hospitalization). Sociodemographic characteristics included age, sex, race and ethnicity, education, marital status, living situation, and income. Because race and ethnicity are key sociodemographic characteristics and known determinants of health outcomes, information on race and ethnicity was obtained from HRS interviews that asked participants to self-identify their race and ethnicity. These data are reported as Hispanic, non-Hispanic Black (hereinafter, Black), non-Hispanic White (hereinafter, White), or non-Hispanic other race. We considered all participants who self-identified as Hispanic to be Hispanic regardless of reported race. We combined participants who reported being American Indian or Alaska Native, Asian or Pacific Islander, or of other race under the “other” category because of small numbers in these categories. Health characteristics included the Lee index score, Charlson Comorbidity Index score, and baseline functional and cognitive status. The Lee index is a freely available prognostic index developed to estimate mortality among community-dwelling older adults. It includes key demographics (age and sex), body mass index (calculated as weight in kilograms divided by height in meters squared), selected comorbidities (cancer, diabetes, chronic obstructive pulmonary disease, active tobacco use, or heart failure), mobility (walking several blocks), strength (ability to pull or push large objects), and functioning (bathing and managing finances) weighted to produce a score (range, 0-26).^17^ Predicted 4-year mortality ranges from 4% for a Lee index score of less than 6, 15% to 42% for scores between 6 and 13, and 64% for scores of 14 or greater. To our knowledge, the Lee index has not been tested for functional and cognitive outcomes among patients with acute illness. Charlson Comorbidity Index scores were derived using *International Classification of Diseases, Ninth Revision* and *Tenth Revision* (*ICD-9* and *ICD-10*), codes from FFS Medicare inpatient claims.

Functional status was ascertained from HRS interviews as the count of dependencies in activities of daily living (ADLs), determined as self-reported need for assistance in walking across a room, dressing, bathing, eating, transferring in or out of bed, or toileting (range, 0-6). Cognitive status was ascertained from HRS interviews using the Langa-Weir algorithm, which uses self-reported and proxy measures to classify participants into 3 groups: normal cognition, cognitive impairment nondementia (CIND), and dementia.^18^ We combined information on functional and cognitive status to define 3 categories of overall impairment: no impairment (no dependency in ADLs and normal cognition), mild impairment (dependency in 1 ADL, CIND, or both), and severe impairment (dependencies in ≥2 ADLs, dementia, or both). Measures of illness severity included the following: length of stay and prolonged intensive care unit (ICU) stay of 3 or more days during acute care hospitalization; receipt of mechanical ventilation during acute care or LTCH hospitalization (*ICD-9* procedure codes 96.04 and 96.7x; *ICD-10* procedure code 5A1955Z; and Centers for Medicare and Medicaid Services LTCH Diagnosis-Related Groups 3, 4, 207, 870, 927, and 933); and tracheostomy (*ICD-9* procedure code 31.1x and 31.2x; *Current Procedural Terminology* codes 31600, 31601, 31603, and 31605; and *ICD-10* code V44.0).

### Outcome

Our primary outcome was death or survival with 2 or more ADL dependencies or dementia, representing a combination of vital status, function, and cognition within 2.5 years of LTCH hospitalization, the time period for biennial HRS interviews. For the few survivors missing a post-LTCH interview (n = 8), we imputed functional and cognitive status from the prehospitalization interview assuming no change. Because the median time to recovery among older ICU survivors is nearly 3 months,^19^ we only included assessments conducted 90 days or more after LTCH admission to ascertain outcomes. Death was ascertained from the HRS Tracker File, which determines mortality from next-of-kin interviews supplemented with the National Death Index.^20^ We additionally searched the Medicare Master Beneficiary Summary File for completeness of vital status information.

### Statistical Analysis

We examined baseline characteristics of participants, stratified by primary outcome. We described outcomes by pre-LTCH baseline impairment status through a Sankey diagram representing the proportion of participants in each outcome category. Given our small sample size and event rate, we constructed a parsimonious multivariable logistic regression model to estimate associations between a priori selected risk factors available to clinicians and the primary outcome. Candidate factors identified from prior literature and the interdisciplinary expertise of our group included baseline impairment status, Lee prognostic index score, prolonged ICU stay, and mechanical ventilation during acute care or LTCH hospitalization.^5,10^ To assess the relative contribution of factors, we estimated a population attributable fraction. To facilitate shared decision-making and care planning for the subgroup of patients with a prolonged ICU stay and mechanical ventilation, we created a nomogram of estimated probability of an adverse outcome for factors with an association. We selected this subgroup because it is most representative of the LTCH population since implementation of the Centers for Medicare & Medicaid Services (CMS) site-neutral payment policy in 2016 that stipulates prolonged ICU stay, use of mechanical ventilation, or both for full reimbursement for LTCH hospitalizations.^21^

We conducted several sensitivity analyses to examine the robustness of our primary modeling approach. First, since we hypothesized that participants with severe impairment at baseline would be unlikely to improve after hospitalization, we repeated our analyses for the outcome of death only. Second, because functional recovery can occur up to 6 months after critical illness, we conducted a sensitivity analysis excluding those who completed their post-LTCH interview before this period. Third, since the measure of dependency in bathing in the Lee index overlaps with functional impairment in ADLs,^17^ we repeated our multivariable model with a modified version of the Lee index omitting this factor. Fourth, given the variability in time between hospitalization and post-LTCH HRS interviews, we repeated our models accounting for time as a covariate. Fifth, because survey weights may not yield a nationally representative sample for our cohort,^22^ we repeated our primary models using unweighted data. For all analyses, unless otherwise specified, we accounted for the complex survey design of the HRS and generated weighted means and percentages. *P* < .05 (2-tailed) was considered significant in all analyses. Analyses were performed in Stata, version 14 (StataCorp LLC), and SAS, version 9.4 (SAS Institute Inc). Data were analyzed between November 1, 2021, and June 30, 2023.

## Results

We identified 396 participants hospitalized across 167 LTCHs between 2003 and 2016 (Figure 1). Their median age was 75 (IQR, 68-82) years; 201 (51%) were women and 195 (49%) were men (Table 1). There were 84 participants (15%) who identified as Black, 59 (12%) as Hispanic or of other race, and 253 (74%) as White. A total of 109 participants (24%) were dually enrolled in Medicare and Medicaid, and 125 (28%) had severe impairment at baseline. Half (212 [54%]) had a prolonged ICU stay and 153 (41%) received mechanical ventilation during acute care or LTCH hospitalization. The most common LTCH diagnosis was respiratory illness (139 [38%]).

**Table 1.** Baseline Characteristics of Study Participants^a^

Characteristic	Overall cohort (N = 396)	Death or severe impairment within 2.5 y after LTCH hospitalization	
		No (n = 78)	Yes (n = 318)
Sociodemographic			
Age, median (IQR), y	75 (68-82)	71 (66-77)	76 (70-83)
Sex			
Female	201 (51)	38 (48)	163 (52)
Male	195 (49)	40 (52)	155 (48)
Race and ethnicity^b^			
Hispanic or non-Hispanic other race^c^	59 (12)	NA	NA
Non-Hispanic Black	84 (15)	NA	NA
Non-Hispanic White	253 (74)	43 (67)	210 (75)
Education: less than high school	173 (38)	29 (31)	144 (40)
Marital status: married or partnered	182 (46)	42 (59)	140 (42)
Living situation: living alone	127 (34)	29 (34)	98 (34)
Income, median (IQR), per $1000	21.0 (11.3-38.4)	25.9 (14.4-54.8)	19.5 (10.8-34.9)
Dual Medicare and Medicaid enrollment	109 (24)	NA	NA
Prehospitalization health			
Self-rated health			
Excellent, very good, or good	139 (35)	36 (44)	103 (34)
Fair or poor	257 (65)	42 (56)	215 (67)
Lee index score, median (IQR)^d^	10 (8-14)	8 (6-10)	11 (9-14)
CCI score, median (IQR)	2 (1-3)	2 (1-3)	2 (1-4)
Comorbidity			
Diabetes	165 (40)	NA	NA
Cancer	78 (20)	NA	NA
COPD	97 (29)	NA	NA
Congestive heart failure	184 (49)	27 (40)	157 (51)
Smoking	47 (15)	NA	NA
No. of ADL dependencies			
0	251 (65)	70 (90)	181 (57)
1	46 (13)	NA	NA
≥2	99 (22)	NA	NA
Cognitive impairment			
No impairment	199 (56)	54 (69)	145 (46)
CIND	129 (30)	NA	NA
Dementia	68 (14)	NA	NA
Overall impairment^e^			
No	147 (40)	48 (64)	99 (34)
Mild	124 (32)	24 (28)	100 (33)
Severe	125 (28)	NA	NA
Hospitalization before LTCH transfer			
Length of stay, median (IQR), d	11 (7-20)	10 (6-18)	12 (7-20)
Prolonged ICU stay (≥3 d)	212 (54)	36 (55)	176 (62)
LTCH hospitalization			
Length of stay, median (IQR), d	23 (16-32)	22 (17-28)	24 (16-33)
Respiratory discharge diagnosis^f^	139 (38)	NA	NA
Tracheostomy	61 (19)	NA	NA
Met criteria for full reimbursement^g^	216 (54)	36 (50)	180 (55)
Any mechanical ventilation during acute care or LTCH stay	153 (41)	28 (39)	125 (42)

Abbreviations: ADL, activity of daily living; CCI, Charlson Comorbidity Index; CIND, cognitive impairment nondementia; COPD, chronic obstructive pulmonary disease; HRS, Health and Retirement Study; ICU, intensive care unit; LTCH, long-term acute care hospital; NA, not available (data not shown for cell sizes ≤25).

^a^ Unless indicated otherwise, values are presented as the unweighted No. (weighted %) of participants.

^b^ Obtained from HRS interviews that asked participants to self-identify their race and ethnicity.

^c^ All who self-identified as Hispanic were considered Hispanic regardless of reported race. Due to small numbers, the “other” category combines participants who reported being American Indian or Alaska Native, Asian or Pacific Islander, or of other race.

^d^ Prognostic index for survival developed and validated using the HRS cohort, which stratifies adults aged 50 years or older into risk groups (high, intermediate, or low) for 4-year mortality (scores estimate risk as follows: 0-5, <4% risk; 6-9, 15% risk; 10-13, 42% risk; and ≥14, 64% risk).

^e^ Defined by a combination of functional and cognitive status as detailed in the Methods, and categorized as no impairment (no dependency in ADLs and normal cognition), mild impairment (dependency in 1 ADL, CIND, or both), or severe impairment (dependencies in ≥2 ADLs, dementia, or both).

^f^ As determined by Major Diagnostic Category code 4 and Centers for Medicare and Medicaid Services LTCH Diagnosis-Related Group codes 3 and 4.

^g^ Patients who (1) spend 3 days or more in an ICU during the preceding acute care hospitalization, (2) require prolonged mechanical ventilation of 96 hours or longer, or (3) have a primary LTCH diagnosis other than psychiatric or rehabilitation.

In terms of the primary outcome, 318 participants (79%) died or survived with severe impairment. The median time to death was 94 (IQR, 29-306) days after LTCH admission; 63 participants (25%) died during LTCH hospitalization and another 58 (23%) died within 90 days of discharge. Participants who died or survived with severe impairment were older (median age, 76 [IQR, 70-83] vs 71 [66-77] years) and had lower income (median, $19 474 [IQR, $10 800-$34 928] vs $25 930 [$14 400-$54 765]) than those who survived with no or mild impairment. During the acute care hospitalization preceding LTCH transfer, participants who died or survived with severe impairment had a longer hospital length of stay (median, 12 [IQR, 7-20] vs 10 [6-18] days) and more frequently had a prolonged ICU stay (176 [62%] vs 36 [55%]).

**Figure 2.**
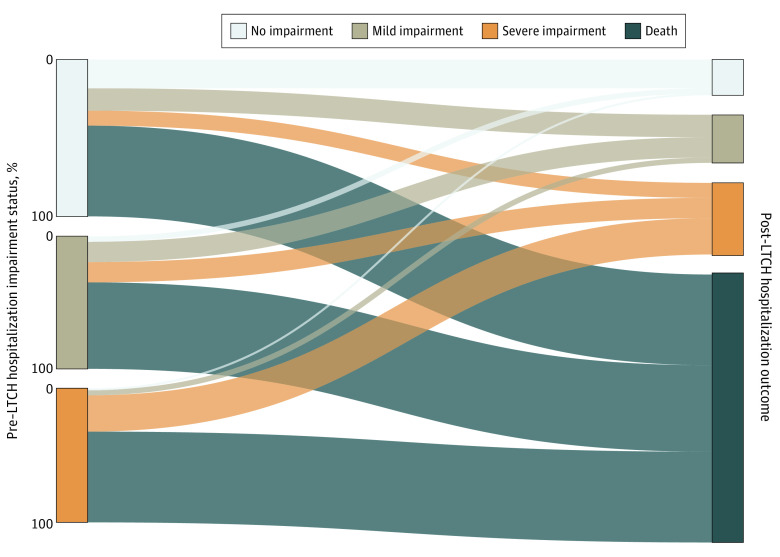
Proportions of Participants Across Categories of Impairment and Survival After Long-Term Acute Care Hospital (LTCH) Hospitalization by Pre-LTCH Impairment Status Weighted percentages are reported for all categories. For the smallest categories of transitions, the values are as follows: 5%, from mild to no impairment; 1%, from severe to no impairment; and 5%, from severe to mild impairment. No impairment indicates no dependency in activities of daily living (ADLs) and normal cognition; mild impairment indicates dependency in 1 ADL, cognitive impairment nondementia, or both; and severe impairment indicates dependencies in 2 or more ADLs, dementia or both.

Outcomes varied substantially by baseline impairment status (Figure 2). Fewer participants without pre-LTCH impairment (99 [67%]) died or survived with severe impairment after hospitalization than those with mild (100 [81%]) or severe impairment (118 [94%]). In sensitivity analysis evaluating death only, 85 participants (58%) without pre-LTCH impairment died within 2.5 years of LTCH admission compared with 85 participants (70%) with severe impairment (eFigure 1 in Supplement 1). When we excluded the 215 participants who completed the post-LTCH interview within 6 months of hospitalization and may not have recovered maximally, proportions of an adverse outcome stratified by pre-LTCH impairment status were not substantively different from the main analysis (eFigure 2 in Supplement 1).

In multivariable analysis, a 5-point increase in the Lee index score, signifying worse survival prognosis, was associated with 3-fold greater odds of experiencing death or severe impairment within 2.5 years of an LTCH hospitalization (adjusted odds ratio [AOR], 3.2 [95% CI, 1.7 to 6.0]). Severe impairment before hospitalization was associated with more than 4-fold greater odds (AOR, 4.5 [95% CI, 1.3 to 15.4]) of that outcome (Table 2). Death or severe impairment after LTCH hospitalization was more attributable to baseline health status than severity of acute illness, given population attributable fractions of 19.3% (95% CI, 8.2% to 29.0%) for pre-LTCH Lee index score and 8.1% (95% CI, −0.1% to 15.6%) for pre-LTCH functional and cognitive impairment status compared with 4.3% (95% CI, −2.2% to 10.5%) for prolonged ICU stay and 2.7% (95% CI, −2.0% to 7.2%) for receipt of mechanical ventilation (eTable 1 in Supplement 1).

**Table 2.** Multivariable Analysis Identifying Factors Associated With Death or Severe Impairment After LTCH Hospitalization

Characteristic	Weighted OR (95% CI)
Baseline functional and cognitive status^a^	
No impairment	1 [Reference]
Mild impairment	1.7 (0.7 to 3.8)
Severe impairment^b^	4.5 (1.3 to 15.4)
Prolonged ICU stay^c^	
No	1 [Reference]
Yes	1.6 (0.8 to 3.1)
Mechanical ventilation in LTCH or preceding acute care hospitalization	
No	1 [Reference]
Yes	1.4 (0.8 to 2.6)
Lee index score (per 5-point increase)^d^	3.2 (1.7 to 6.0)

Abbreviations: ADL, activity of daily living; CIND, cognitive impairment nondementia; HRS, Health and Retirement Study; ICU, intensive care unit; LTCH, long-term acute care hospital; OR, odds ratio.

^a^ Determined from functional and cognitive status ascertained in the HRS interview immediately preceding LTCH hospitalization and categorized as follows: no impairment (no dependency in ADLs and normal cognition), mild impairment (dependency in 1 ADL, CIND, or both), or severe impairment (dependencies in ≥2 ADLs, dementia, or both).

^b^ Includes functional impairment, cognitive impairment, or both.

^c^ Defined as a prolonged ICU stay (≥3 days) during acute care hospitalization before transfer to an LTCH.

^d^ Prognostic index for survival developed and validated using the HRS cohort that stratifies adults aged 50 years or older into risk groups (high, intermediate, or low) for 4-year mortality (scores estimate risk as follows: 0-5, <4% risk; 6-9, 15% risk; 10-13, 42% risk; and ≥14, 64% risk).

In the sensitivity analysis evaluating death only, the association with the Lee index was slightly attenuated (AOR, 2.8 [95% CI, 1.8 to 4.5]). Severe pre-LTCH impairment was no longer associated with death (eTable 2 in Supplement 1). When we excluded participants who completed the post-LTCH interview before 6 months after hospitalization, the magnitude of the association with the Lee index was similar; however, severe impairment was no longer associated with death or severe impairment (eTable 3 in Supplement 1). In the remaining sensitivity analyses removing the overlapping measure of bathing from the Lee index, accounting for time between HRS interview and outcome assessment, and using unweighted data, the associations were similar compared with the main analysis (eTables 4, 5, and 6 in Supplement 1).

**Figure 3.**
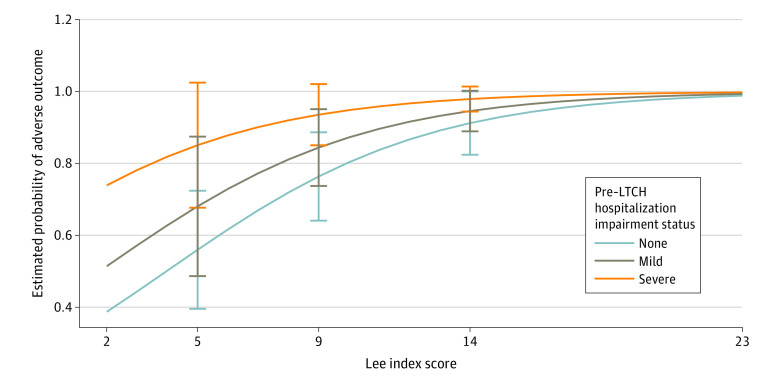
Estimated Probability of Death or Severe Impairment After Long-Term Acute Care Hospital (LTCH) Hospitalization by Baseline Lee Index Prognostic Score and Pre-LTCH Impairment Status Among Participants Who Received Mechanical Ventilation We selected this subgroup because it is most representative of the current LTCH population since implementation of the prospective payment system in 2016 that requires prolonged intensive care unit (ICU) stay, use of mechanical ventilation, or both for reimbursement of admission to an LTCH. Severe impairment includes functional impairment, cognitive impairment, or both. Error bars indicate 95% CIs.

To facilitate interpretation of the relative contribution of factors in our multivariable model, we estimated the probability of an adverse outcome for a given Lee index score across different baseline impairment status for participants with a prolonged ICU stay and mechanical ventilation (Figure 3). Although the prevalence of an adverse outcome was high in this population overall, the probability was lower for patients with good baseline survival prognosis (Lee index <6) and no or mild impairment (range, 56%-68%). For those with exceedingly poor survival prognosis (Lee index ≥14), the probability of an adverse outcome ranged from 91% to 98% and did not vary meaningfully by baseline impairment status.

## Discussion

In this cohort study of middle-aged and older US adults enrolled in a nationally representative survey, we found that 4 of 5 participants died or survived with severe impairment, defined as dependencies in 2 or more ADLs or dementia within 2.5 years of hospitalization in an LTCH. The probability of dying or surviving with severe impairment was highly dependent on health status before admission. The patients most likely to survive with reasonably intact function and cognition were those who had a good survival prognosis before admission (Lee index <6) with either no or mild impairment (≤1 ADL dependency and no dementia). Our findings highlight the importance of factoring in preillness health status to inform goals of care and decision-making for older adults with prolonged acute illness.

Previous investigations focused on older adults in LTCHs reported survival but not long-term functional and cognitive outcomes.^4,5,6^ Among studies including adults across the lifespan,^8,10,11,12,23^ good functional status has been reported for as many as 50% of adults^12^ and no or mild cognitive impairment in more than three-fourths^11^ in the year after LTCH hospitalization. In contrast, we observed a greater prevalence of death or survival with severe impairment (functional, cognitive, or both) in our cohort. This discrepancy is not surprising because prior prospective studies included patients who were younger and relatively functionally intact at baseline, excluding up to four-fifths of eligible participants and potentially yielding overly optimistic estimates.^24^ Because our study sample was derived from a longitudinal survey that prospectively assessed functional and cognitive status before and after LTCH hospitalization, we were able to characterize the composite outcome of survival, function, and cognition in a population of older adults who often have preexisting impairments.

Our findings have important clinical implications. First, the observation that 4 in 5 older adults died or survived with severe impairment (functional, cognitive, or both) after LTCH hospitalization highlights the near universal need for palliative care and discussion of goals of care in this population. A previous study reported that among Medicare beneficiaries hospitalized in an LTCH, only 1% received a specialist palliative care consultation during the acute care hospitalization preceding transfer or in the LTCH.^5^ Although LTCHs provide multidisciplinary care, including a focus on ventilator weaning and interdisciplinary rehabilitation, only one-third offer palliative care.^3,25^ Therefore, conversations about goals of care and involvement of specialist palliative expertise should occur during the acute care hospitalization preceding LTCH transfer. Second, the risk factors identified in our study can be used by clinicians to guide shared decision-making by providing a range of probabilities of a composite adverse outcome that older adults and their caregivers value more in treatment decisions and care planning than survival alone.^26^ For example, a 76-year-old male smoker with chronic lung disease and dependency in bathing and managing finances at baseline (Lee index = 14) is unlikely to survive without severe impairments after LTCH hospitalization. Thus, the care team should share this prognostic information and discuss hospice as an alternative to prolonged hospital care in an LTCH if aligned with the goals of both the patient and the caregiver. In contrast, a 66-year-old female nonsmoker with diabetes, no or mild cognitive impairment, and no dependency in ADLs but difficulty managing finances (Lee index = 5) has a 30% to 50% probability of surviving without severe functional and cognitive impairment. Depending on the goals of care, this patient could benefit from prolonged acute care in an LTCH setting. Our results suggest that assessing survival prognosis using the Lee index (available online^27^), as well as ADL functioning and cognitive status before acute illness, can inform expectations and guide treatment choices among older adults with prolonged acute illness.

Our study has several notable strengths that advance our understanding of prognosis after a prolonged acute illness. First, our findings are derived from a national multicenter sample of older US adults, in contrast with prior studies that were limited to a single site or a few centers.^8,10,11,12,28^ As such, our cohort had substantial representation of Black adults and those from lower socioeconomic backgrounds not commonly represented in prospective recovery cohorts of prolonged acute illness.^11,12,23^ Second, we included all patients with an LTCH hospitalization and prehospitalization HRS assessments, which circumvents issues of recall bias of prior health status and selection bias of including healthier patients who are more likely to consent to participate. Third, our observed rates of survival are consistent with prior literature, which provides external validity to our findings.^6,8,10,23^ Fourth, we examined functional and cognitive outcomes in addition to survival after an LTCH stay, patient-centered outcomes that matter to older adults.^29^

### Limitations

Our findings should be interpreted in the context of certain limitations. First, our assessment of illness severity was limited to characteristics that could be ascertained in claims records. Nevertheless, we included a prolonged ICU stay and mechanical ventilation in our models, 2 clinical factors known to be important in prior work examining functional outcomes after acute and critical illness.^5,30,31^ Second, our study included patients with an LTCH hospitalization before implementation of the CMS site-neutral payment policy in 2016, designed to narrow LTCH focus to more severely ill patients with a prolonged ICU stay or mechanical ventilation. Thus, we anticipate the risk of an adverse outcome in a contemporary LTCH population to be even worse than estimates obtained in our study. Third, the relatively small cohort precluded evaluation of the following: individual components of the Lee index as risk factors; functional and cognitive impairments as separate outcomes; outcomes associated with LTCH facility-level characteristics; or disentanglement of outcomes by different care pathways after the LTCH stay, including postacute care delivered in skilled nursing facilities or rehabilitation hospitals or at home via home health services.^5,32^ Finally, although we used weights in our analyses as recommended by the HRS, our findings may not be nationally representative due to the relatively small sample size and to potential for differences in weights for participants with linked vs unlinked Medicare data.^22^ The similar estimates for risk factors in our unweighted models, however, increase confidence in our findings.

## Conclusions

In this nationally representative cohort study of middle-aged and older adults admitted to an LTCH, 4 of 5 participants died or survived with severe impairment (functional, cognitive, or both) within 2.5 years after admission. Individuals with favorable baseline survival prognosis (Lee index <6) and no or mild functional and cognitive impairment were most likely to survive with reasonably intact function and cognition. Our study highlights that baseline survival prognosis and functional and cognitive status should be considered in decision-making for middle-aged and older adults and their caregivers faced with the decision of prolonged acute care.

## Notes

Supplement 1. **eTable 1.** Population Attributable Fraction (PAF) for Factors Evaluated in the Multivariable Model for the Outcome of Death or Severe Impairment After Long-Term Acute Care Hospitalization **eTable 2.** Multivariable Analysis Identifying Factors Associated With the Outcome of Death Following LTCH Hospitalization **eTable 3.** Multivariable Analysis Identifying Factors Associated With Death or Severe Impairment Following Hospitalization in an LTCH Among the Subgroup of Participants With Post-LTCH Interview or Death Beyond 6 Months of Hospitalization (n = 215) **eTable 4.** Sensitivity Analysis Replacing the Lee Index in the Multivariable Model With a Modified Version Removing the Factor of Dependency in Bathing That Overlaps With the Assessment of Functional Impairment **eTable 5.** Sensitivity Analysis With Additional Adjustment for Time Between Admission to an LTCH and Assessment of the Outcome of Either Death or Survival With Severe Impairment as Assessed in the Post-LTCH Health and Retirement Study (HRS) Interview **eTable 6.** Sensitivity Analysis Repeating the Multivariable Model for the Outcome of Death or Survival With Severe Impairment Without Survey Weights **eFigure 1.** Post-LTCH Outcome of Death Only, Stratified by Pre-LTCH Admission Impairment Status **eFigure 2.** Post-LTCH Outcome Stratified by Pre-LTCH Impairment Status Among the Subgroup of Participants Who Completed the Post-LTCH HRS Interview or Died Beyond 6 Months of Hospital Discharge (n = 215)

Supplement 2.
Data Sharing Statement
