# Supplementary material for: Survival, Function, and Cognition After Hospitalization in Long-Term Acute Care Hospitals
Source: JAMA Netw Open. 2024 May 28;7(5):e2413309. doi: 10.1001/jamanetworkopen.2024.13309 (PMC11134219; doi:10.1001/jamanetworkopen.2024.13309)
Supplement: Supplement 1. — eTable 1. Population Attributable Fraction (PAF) for Factors Evaluated in the Multivariable Model for the Outcome of Death or Severe Impairment After Long-Term Acute Care Hospitalization eTable 2. Multivariable Analysis Identifying Factors Associated With the Outcome of Death Following LTCH Hospitalization eTable 3. Multivariable Analysis Identifying Factors Associated With Death or Severe Impairment Following Hospitalization in an LTCH Among the Subgroup of Participants With Post-LTCH Interview or Death Beyond 6 Months of Hospitalization (n = 215) eTable 4. Sensitivity Analysis Replacing the Lee Index in the Multivariable Model With a Modified Version Removing the Factor of Dependency in Bathing That Overlaps With the Assessment of Functional Impairment eTable 5. Sensitivity Analysis With Additional Adjustment for Time Between Admission to an LTCH and Assessment of the Outcome of Either Death or Survival With Severe Impairment as Assessed in the Post-LTCH Health and Retirement Study (HRS) Interview eTable 6. Sensitivity Analysis Repeating the Multivariable Model for the Outcome of Death or Survival With Severe Impairment Without Survey Weights eFigure 1. Post-LTCH Outcome of Death Only, Stratified by Pre-LTCH Admission Impairment Status eFigure 2. Post-LTCH Outcome Stratified by Pre-LTCH Impairment Status Among the Subgroup of Participants Who Completed the Post-LTCH HRS Interview or Died Beyond 6 Months of Hospital Discharge (n = 215) [file jamanetwopen-e2413309-s001.pdf]

## Supplementary Online Content

Jain S, Gan S, Nguyen OK, et al. Survival, function, and cognition after hospitalization in long-term acute care hospitals. *JAMA Netw Open*. 2024;7(5):e2413309. doi:10.1001/jamanetworkopen.2024.13309

**eTable 1.** Population Attributable Fraction (PAF) for Factors Evaluated in the Multivariable Model for the Outcome of Death or Severe Impairment After Long-Term Acute Care Hospitalization

**eTable 2.** Multivariable Analysis Identifying Factors Associated With the Outcome of Death Following LTCH Hospitalization

**eTable 3.** Multivariable Analysis Identifying Factors Associated With Death or Severe Impairment Following Hospitalization in an LTCH Among the Subgroup of Participants With Post-LTCH Interview or Death Beyond 6 Months of Hospitalization (n = 215)

**eTable 4.** Sensitivity Analysis Replacing the Lee Index in the Multivariable Model With a Modified Version Removing the Factor of Dependency in Bathing That Overlaps With the Assessment of Functional Impairment

**eTable 5.** Sensitivity Analysis With Additional Adjustment For Time Between Admission to an LTCH and Assessment of the Outcome of Either Death or Survival With Severe Impairment as Assessed in the Post-LTCH Health and Retirement Study (HRS) Interview

**eTable 6.** Sensitivity Analysis Repeating the Multivariable Model for the Outcome of Death or Survival With Severe Impairment Without Survey Weights

**eFigure 1.** Post-LTCH Outcome of Death Only, Stratified by Pre-LTCH Admission Impairment Status

**eFigure 2.** Post-LTCH Outcome Stratified by Pre-LTCH Impairment Status Among the Subgroup of Participants Who Completed the Post-LTCH HRS Interview or Died Beyond 6 Months of Hospital Discharge (n = 215)

This supplementary material has been provided by the authors to give readers additional information about their work.

© 2024 Jain S et al. *JAMA Network Open.*

**eTable 1.** Population Attributable Fraction (PAF) for Factors Evaluated in the Multivariable Model for the Outcome of Death or Severe Impairment After Long-Term Acute Care Hospitalization

To calculate the PAF, the value of each factor was set to best case scenario – 1) baseline functional and cognitive status: 0 ADL dependency & normal cognition; 2) prolonged ICU stay: no; 3) mechanical ventilation: no; and 4) Lee prognostic index: 6.

| Predictor | Population Attributable Fraction (95% CI) |
|---|---|
| Baseline functional and cognitive status<br>*if all participants had no impairment vs current cohort | 8.1% (-0.1%, 15.6%) |
| Prolonged ICU stay<br>*if all participants had no prolonged ICU stay vs current cohort | 4.3% (-2.2%, 10.5%) |
| Mechanical ventilation in acute care hospital or LTACH<br>*if all participants had no mechanical ventilation vs current cohort | 2.7% (-2.0, 7.2%) |
| Lee prognostic index<br>*If Lee index was 6 vs the current cohort (mean=11) | 19.3% (8.2%, 29.0%) |

© 2024 Jain S et al. *JAMA Network Open.*

**eTable 2.** Multivariable Analysis Identifying Factors Associated With the Outcome of Death Following LTCH Hospitalization

| Characteristic | Weighted OR (95% CI) |
|---|---|
| [a]Pre-hospitalization functional and cognitive status | |
| No impairment | ref. |
| Mild impairment | 1.11 (0.53, 2.31) |
| Severe impairment | 0.89 (0.42, 1.92) |
| [b]Prolonged ICU stay | |
| No | ref. |
| Yes | 1.78 (0.95, 3.29) |
| Mechanical ventilation in LTCH or preceding acute care hospitalization | |
| No | ref. |
| Yes | 1.47 (0.92, 2.37) |
| [c]Lee index (per 5-point increase) | 2.83 (1.80, 4.46) |

ICU = Intensive Care Unit; OR = Odds Ratio; LTCH = Long-Term Acute Care Hospital
[a]Determined from functional and cognitive status ascertained in HRS interview immediately preceding LTCH hospitalization and categorized as 1) No impairment: no dependency in activities of daily living (ADLs) and normal cognition; 2) Mild impairment: dependency in 1 ADL or cognitive impairment non-dementia (CIND); and 3) Severe impairment: dependency in ≥2 ADLs or dementia.
[b] Defined as a stay of ≥3 days in the ICU during the acute care hospitalization prior to transfer to LTCH.
[c] Lee index is a prognostic index developed and validated using the Health and Retirement Study cohort that stratifies adults ≥50 years into high-, intermediate-, and low-risk groups for 4-year mortality. A score of 0-5 predicts a less than 4% risk, 6-9 a 15% risk, 10-13 a 42% risk, and ≥14 a 64% risk of 4-year mortality.

© 2024 Jain S et al. *JAMA Network Open.*

**eTable 3.** Multivariable Analysis Identifying Factors Associated With Death or Severe Impairment Following Hospitalization in an LTCH Among the Subgroup of Participants With Post-LTCH Interview or Death Beyond 6 Months of Hospitalization (n = 215)

| Characteristic | Weighted OR (95% CI) |
|---|---|
| [a]Pre-hospitalization functional and cognitive status | |
| No impairment | ref. |
| Mild impairment | 1.63 (0.72, 3.68) |
| Severe impairment | 3.67 (0.98, 13.78) |
| [b]Prolonged ICU stay | |
| No | ref. |
| Yes | 1.26 (0.58, 2.76) |
| Mechanical ventilation in LTCH or preceding acute care hospitalization | |
| No | ref. |
| Yes | 0.89 (0.42, 1.90) |
| [c]Lee index (per 5-point increase) | 2.92 (1.41, 6.07) |

ICU = Intensive Care Unit; OR = Odds Ratio; LTCH = Long-Term Acute Care Hospital
[a]Determined from functional and cognitive status ascertained in HRS interview immediately preceding LTCH hospitalization and categorized as 1) No impairment: no dependency in activities of daily living (ADLs) and normal cognition; 2) Mild impairment: dependency in 1 ADL or cognitive impairment non-dementia (CIND); and 3) Severe impairment: dependency in ≥2 ADLs or dementia.
[b] Defined as a stay of ≥3 days in the ICU during the acute care hospitalization prior to transfer to LTCH.
[c] Lee index is a prognostic index developed and validated using the Health and Retirement Study cohort that stratifies adults ≥50 years into high-, intermediate-, and low-risk groups for 4-year mortality. A score of 0-5 predicts a less than 4% risk, 6-9 a 15% risk, 10-13 a 42% risk, and ≥14 a 64% risk of 4-year mortality.

© 2024 Jain S et al. *JAMA Network Open.*

**eTable 4.** Sensitivity Analysis Replacing the Lee Index in the Multivariable Model With a Modified Version Removing the Factor of Dependency in Bathing That Overlaps With the Assessment of Functional Impairment

| Characteristic | Weighted OR (95% CI) |
|---|---|
| [a]Pre-hospitalization functional and cognitive status | |
| No impairment | ref. |
| Mild impairment | 1.77 (0.77, 4.09) |
| Severe impairment | 6.03 (1.74, 20.88) |
| [b]Prolonged ICU stay | |
| No | ref. |
| Yes | 1.57 (0.80, 3.07) |
| Mechanical ventilation in LTCH or preceding acute care hospitalization | |
| No | ref. |
| Yes | 1.44 (0.77, 2.68) |
| [c]Modified Lee index (per 5-point increase) | 3.16 (1.70, 5.88) |

ICU = Intensive Care Unit; OR = Odds Ratio; LTCH = Long-Term Acute Care Hospital
[a]Determined from functional and cognitive status ascertained in HRS interview immediately preceding LTCH hospitalization and categorized as 1) No impairment: no dependency in activities of daily living (ADLs) and normal cognition; 2) Mild impairment: dependency in 1 ADL or cognitive impairment non-dementia (CIND); and 3) Severe impairment: dependency in ≥2 ADLs or dementia.
[b] Defined as a stay of ≥3 days in the ICU during the acute care hospitalization prior to transfer to LTCH.
[c] Lee index is a prognostic index developed and validated using the Health and Retirement Study cohort that stratifies adults ≥50 years into high-, intermediate-, and low-risk groups for 4-year mortality. A score of 0-5 predicts a less than 4% risk, 6-9 a 15% risk, 10-13 a 42% risk, and ≥14 a 64% risk of 4-year mortality. The version used in this model calculates Lee index score excluding the factor of dependency in bathing that overlaps with assessment of ADLs factored into pre-hospitalization functional and cognitive status.

© 2024 Jain S et al. *JAMA Network Open.*

**eTable 5.** Sensitivity Analysis With Additional Adjustment For Time Between Admission to an LTCH and Assessment of the Outcome of Either Death or Survival With Severe Impairment as Assessed in the Post-LTCH Health and Retirement Study (HRS) Interview

| Characteristic | Weighted OR (95% CI) |
|---|---|
| [a]Baseline functional and cognitive status | |
| No impairment | ref. |
| Mild impairment | 1.65 (0.71, 3.86) |
| Severe impairment | 4.17 (1.15, 15.13) |
| [b]Prolonged ICU stay | |
| No | ref. |
| Yes | 1.11 (0.55, 2.22) |
| Mechanical ventilation in LTCH or preceding acute care hospitalization | |
| No | ref. |
| Yes | 1.04 (0.53, 2.04) |
| [c]Lee index (per 5-point increase) | 3.03(1.70, 5.41) |

Observations with missing post-LTCH HRS interviews for survivors (n=8) were excluded from this analysis.
ICU = Intensive Care Unit; OR = Odds Ratio; LTCH = Long-Term Acute Care Hospital
[a]Determined from functional and cognitive status ascertained in HRS interview immediately preceding LTCH hospitalization and categorized as 1) No impairment: no dependency in activities of daily living (ADLs) and normal cognition; 2) Mild impairment: dependency in 1 ADL or cognitive impairment non-dementia (CIND); and 3) Severe impairment: dependency in ≥2 ADLs or dementia.
[b] Defined as a stay of ≥3 days in the ICU during the acute care hospitalization prior to transfer to LTCH.
[c] Lee index is a prognostic index developed and validated using the Health and Retirement Study cohort that stratifies adults ≥50 years into high-, intermediate-, and low-risk groups for 4-year mortality. A score of 0-5 predicts a less than 4% risk, 6-9 a 15% risk, 10-13 a 42% risk, and ≥14 a 64% risk of 4-year mortality. The version used in this model calculates Lee index score excluding the factor of dependency in bathing that overlaps with assessment of ADLs factored into pre-hospitalization functional and cognitive status.

© 2024 Jain S et al. *JAMA Network Open.*

**eTable 6.** Sensitivity Analysis Repeating the Multivariable Model for the Outcome of Death or Survival With Severe Impairment Without Survey Weights

| Characteristic | Unweighted OR (95% CI) |
|---|---|
| [a]Baseline functional and cognitive status | |
| No impairment | ref. |
| Mild impairment | 1.67 (0.92, 3.06) |
| Severe impairment | 5.30 (2.05, 13.72) |
| [b]Prolonged ICU stay | |
| No | ref. |
| Yes | 1.82 (1.04, 3.19) |
| Mechanical ventilation in LTCH or preceding acute care hospitalization | |
| No | ref. |
| Yes | 1.34 (0.75, 2.38) |
| [c]Lee index (per 5-point increase) | 3.10 (1.97, 4.87) |

ICU = Intensive Care Unit; OR = Odds Ratio; LTCH = Long-Term Acute Care Hospital
[a]Determined from functional and cognitive status ascertained in HRS interview immediately preceding LTCH hospitalization and categorized as 1) No impairment: no dependency in activities of daily living (ADLs) and normal cognition; 2) Mild impairment: dependency in 1 ADL or cognitive impairment non-dementia (CIND); and 3) Severe impairment: dependency in ≥2 ADLs or dementia.
[b] Defined as a stay of ≥3 days in the ICU during the acute care hospitalization prior to transfer to LTCH.
[c] Lee index is a prognostic index developed and validated using the Health and Retirement Study cohort that stratifies adults ≥50 years into high-, intermediate-, and low-risk groups for 4-year mortality. A score of 0-5 predicts a less than 4% risk, 6-9 a 15% risk, 10-13 a 42% risk, and ≥14 a 64% risk of 4-year mortality. The version used in this model calculates Lee index score excluding the factor of dependency in bathing that overlaps with assessment of ADLs factored into pre-hospitalization functional and cognitive status.

© 2024 Jain S et al. *JAMA Network Open.*

**eFigure 1.** Post-LTCH Outcome of Death Only, Stratified by Pre-LTCH Admission Impairment Status

No impairment = no dependency in activities of daily living (ADLs) and normal cognition; mild impairment = dependency in 1 ADL or cognitive impairment non-dementia (CIND); and severe impairment = dependency in ≥2 ADLs or dementia, as described in the methods. LTCH = Long-Term acute Care Hospital.

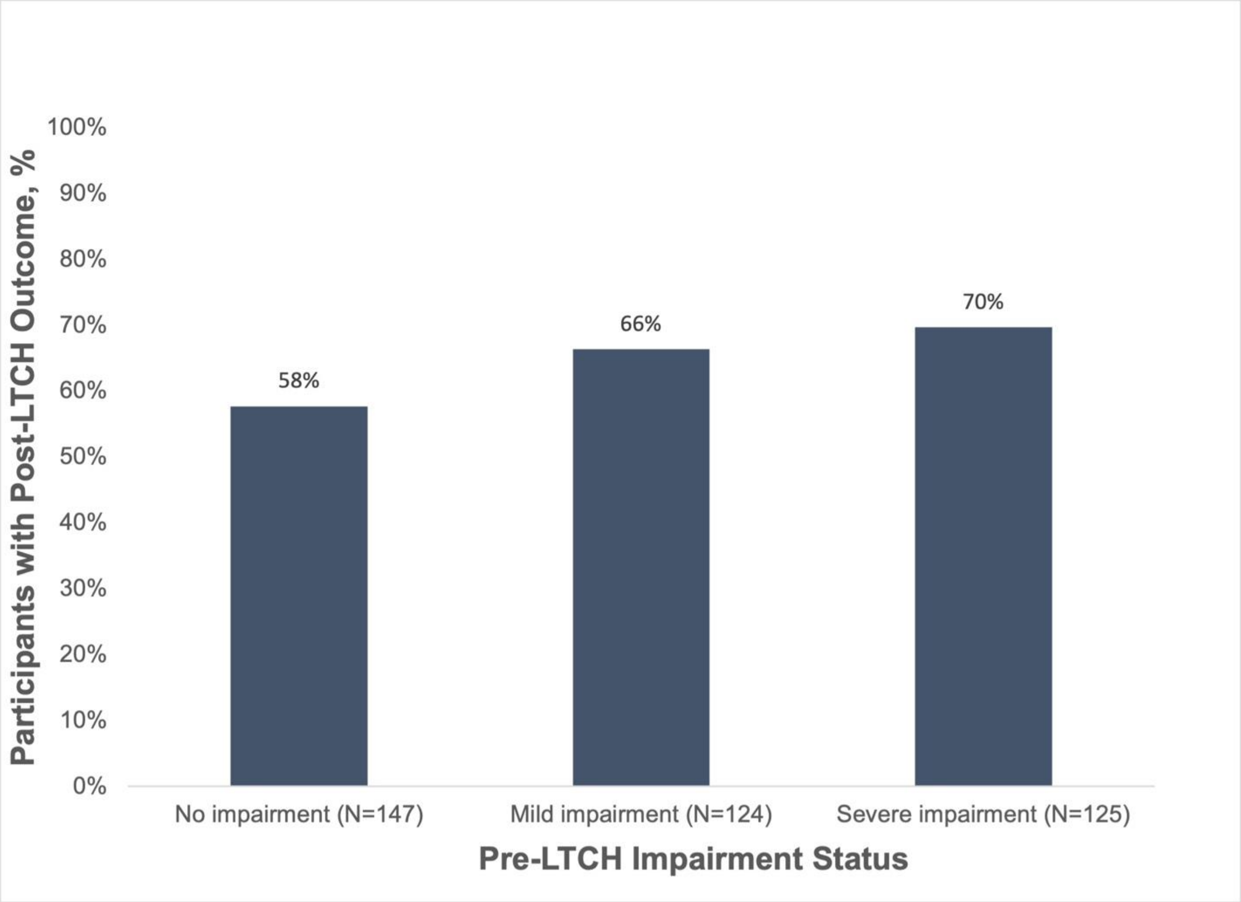


© 2024 Jain S et al. *JAMA Network Open.*

**eFigure 2.** Post-LTCH Outcome Stratified by Pre-LTCH Impairment Status Among the Subgroup of Participants Who Completed the Post-LTCH HRS Interview or Died Beyond 6 Months of Hospital Discharge (n = 215)

No impairment = no dependency in activities of daily living (ADLs) and normal cognition; mild impairment = dependency in 1 ADL or cognitive impairment non-dementia (CIND); and severe impairment = dependency in ≥2 ADLs or dementia, as described in the methods. LTCH = Long-Term acute Care Hospital.

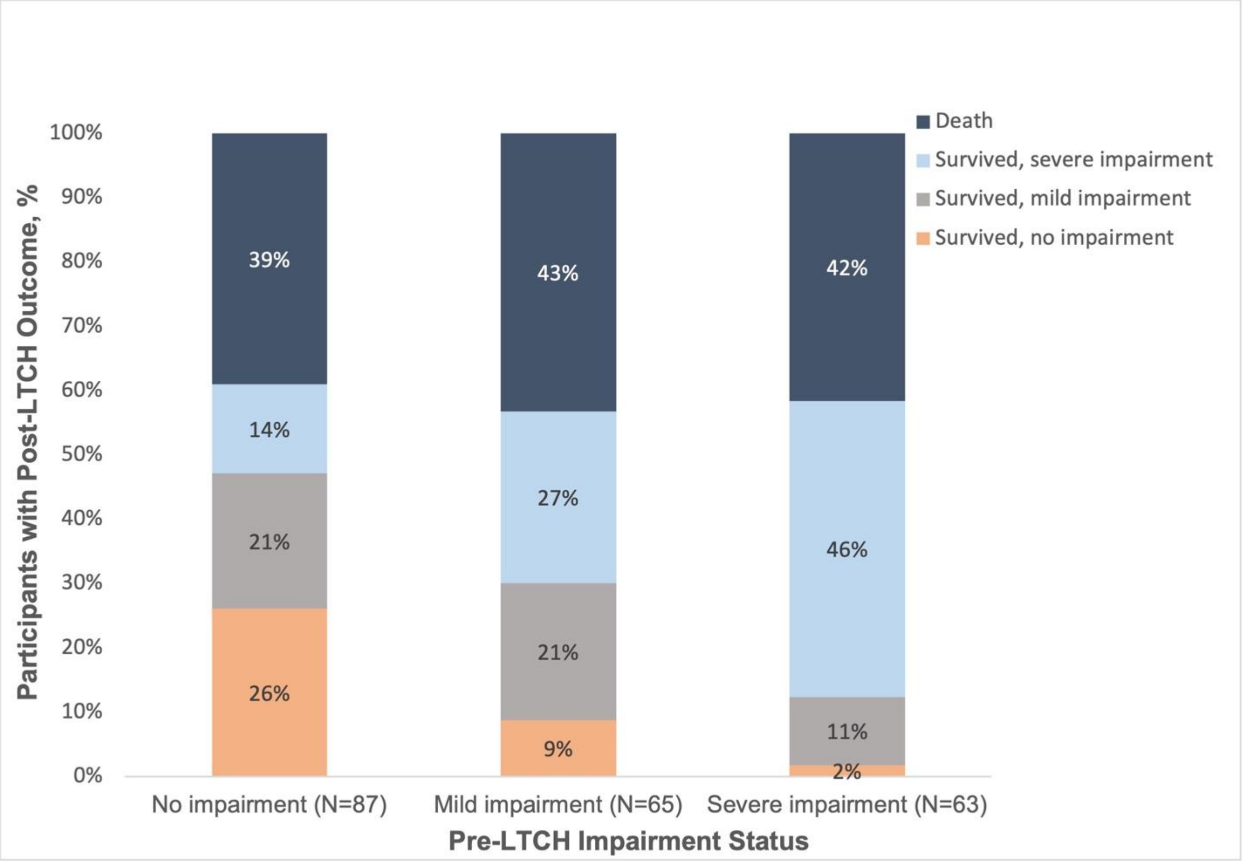


© 2024 Jain S et al. *JAMA Network Open.*
