# Supplementary material for: Survival, Function, and Cognition After Hospitalization in Long-Term Acute Care Hospitals
Source: JAMA Netw Open. 2024 May 28;7(5):e2413309. doi: 10.1001/jamanetworkopen.2024.13309 (PMC11134219; doi:10.1001/jamanetworkopen.2024.13309)
Supplement: Supplement 2. — Data Sharing Statement [file jamanetwopen-e2413309-s002.pdf]

Data Sharing Statement

Jain. Survival, Function, and Cognition After Hospitalization in Long-Term Acute Care Hospitals. *JAMA Netw Open*. Published May 28, 2024.
doi:10.1001/jamanetworkopen.2024.13309

Data
Data available: No

Additional Information
Explanation for why data not available: The data is made available through a data use agreement with the Health and Retirement Study linked with medicare claims
